# Computational and biological evidences on the serotonergic involvement of SeTACN antidepressant-like effect in mice

**DOI:** 10.1371/journal.pone.0187445

**Published:** 2017-11-01

**Authors:** Mariana G. Fronza, Lucimar M. Pinto Brod, Angela Maria Casaril, Manoela Sacramento, Diego Alves, Lucielli Savegnago

**Affiliations:** 1 Programa de Pós Graduação em Biotecnologia, PPGBiotec, Grupo de Pesquisa em Neurobiotecnologia—GPN, CDTec, Universidade Federal de Pelotas, UFPel, Pelotas, RS, Brazil; 2 Programa de Pós Graduação em Química, PPGQ, Laboratório de Síntese Orgânica Limpa—LASOL, CCQFA, Universidade Federal de Pelotas, Pelotas, RS, Brazil; Xi'an Jiaotong University School of Medicine, CHINA

## Abstract

A series of phenylselanyl-1*H*-1,2,3-triazole-4-carbonitriles with different substituents were screened for their binding affinity with serotonin transporter (SERT) and dopamine transporter (DAT) by docking molecular. 5-(4methoxyphenyl)-1-(2-(phenylselanyl)phenyl)-1H-1,2,3-triazole-4-carbonitrile (SeTACN) exhibited the best conformation with SERT even higher than fluoxetine and serotonin, suggesting a competitive inhibition. SeTACN demonstrated additional affinity to other serotonergic receptors involved in antidepressant effects: 5HT_1a_, 5HT_2a_ and 5HT_3_. In another set of experiments, SeTACN led to significant reductions in the immobility time of mice submitted to forced swimming test (FST) in the dose range of 0.1- 20mg/kg, suggesting an antidepressant-like effect. The possible mechanism of action was investigated using serotonergic and dopaminergic antagonists. The antidepressant-like effect of SeTACN (0.1mg/kg i.g.) was prevented by the pretreatment with WAY100635 (a selective 5HT_1a_ antagonist), ketanserin (a 5HT2_a/c_ antagonist) and ondansetron (a selective 5ht_3_ antagonist), PCPA (an inhibitor of serotonin synthesis) but not with SCH23390 (dopaminergic D_1_ antagonist) and sulpiride (D_2_ antagonist). Sub-effective dose of fluoxetine was able to potentiate the effects of a sub-effective dose of SeTACN in FST. None of the treatments affected locomotor activity in open field test (OFT). These results together, suggest that the SeTACN antidepressant-like effect is mediate, at least in parts, by serotonergic system.

## 1. Introduction

Depression is a common, debilitating, life-threatening illness affecting approximately 350 million people worldwide. Despite a huge volume of research in understanding the etiology of depression, the pathophysiological mechanisms involved remain not fully elucidated [1]. Several studies revealed that monoaminergic neurotransmitters, including serotonin (5HT), norepinephrine and dopamine (DA) are the mainly responsible in brain circuits implicated in mood regulation [2, 3]. For this reason, the serotonergic system is one of the most promising targets for the treatment of psychological disorders [4, 5].

Among the antidepressant drugs, the selective serotonin re-uptake inhibitors (SSRI) are most frequently prescribed, due to their higher efficacy, good tolerability and relative safety [6]. On the other hand, the heterogeneity of clinical responses to these drugs and susceptibility to adverse effects still being the antidepressants major clinical problems [7, 8]. However, little progress has been made in decreasing the percentage of resistant cases and improving the antidepressant onset of action [9].

Interestingly, 5HT mediates a wide range of pathways involved in depression through interactions with multiple 5HT receptors. In this context, the flexibility of 5HT system provide a promising opportunity to develop compounds with multiple and complementary modes of action. As the strategy of the simultaneous blocking or stimulation in specific 5HT receptors and/or the SERT inhibition, leading to the blockade of 5HT re-uptake [10, 11]. The adjustment of whole serotonergic transmission via pharmacological agents may provide future alternative antidepressant treatments [12].

Besides the abnormalities in metabolism of neurotransmitters, oxidative stress has been suggested to play an important role in depression pathogenesis [13, 14]. In this perspective, major depressive disorder has been linked to impairments in signaling pathways that regulate neuroplasticity and cell survival [15–17]. In this way, the neuroprotective role of antioxidant compounds can be pharmacologically useful for the modulation of depression [18, 19].

Selenium is an essential trace element nutritionally important to mammals, with physiological roles, in reason of being a structural component of several antioxidant enzymes involved in free radicals decomposition [20–22]. Recently, we reported that a class of phenylselanyl-1H-1,2,3-triazole-4-carbonitriles can induce antioxidant activities in mice cerebral cortex and hippocampus [23].

Several additional studies also demonstrated antidepressant-like activity can be exerted by organoselenium compounds, i.e. (octylseleno)-xylofuranoside [24], α-(phenylselanyl) acetophenone [25], α-phenylselenocitronellal [26], 3-(4-fluorophenylselenyl)-2,5-diphenylselenophene [27] and m-trifluoromethyl-diphenyl diselenide [28]. In parallel, studies have reported that insufficient selenium intake may also affect some psychological roles and the supplementation with selenium was found to be associated with improvements in mood and depression status [29, 30].

In view of the above considerations, the present study reports antidepressant-like analyses of a selenium-containing compound belonging to the class of phenylselanyl-1*H*-1,2,3-triazole-4-carbonitriles. The interaction of this class with 5HT and DA transporters was explored by molecular docking. Based on these results, the affinity with 5TH_1a,_ 5HT_2a_ and 5HT_3_ receptors of 5-(4methoxyphenyl)-1-(2-(phenylselanyl) phenyl)-1H-1,2,3-triazole-4-carbonitrile (SeTACN) was also investigated. As a preliminary biological evaluation, the antidepressant-like effect of SeTACN and the possible mechanism of action was evaluated by behavioral assays in mice.

## 2. Materials and methods

### 2.1 Experimental design

In this study, the affinity with monoamine transporters as SERT and DAT were determined by molecular docking. It was defined as a modelling strategy for further studies involving antidepressant-like potential of the selected compound, since they are the mainly responsible for monoamine clearance from synaptic cleft. In view of extending our knowledge about the mechanism of action performed by the resultant compound, molecular docking in serotonin receptors involved in antidepressant effect: 5TH_1a,_ 5HT_2a_ and 5HT_3_ was also explored.

**Fig 1:**
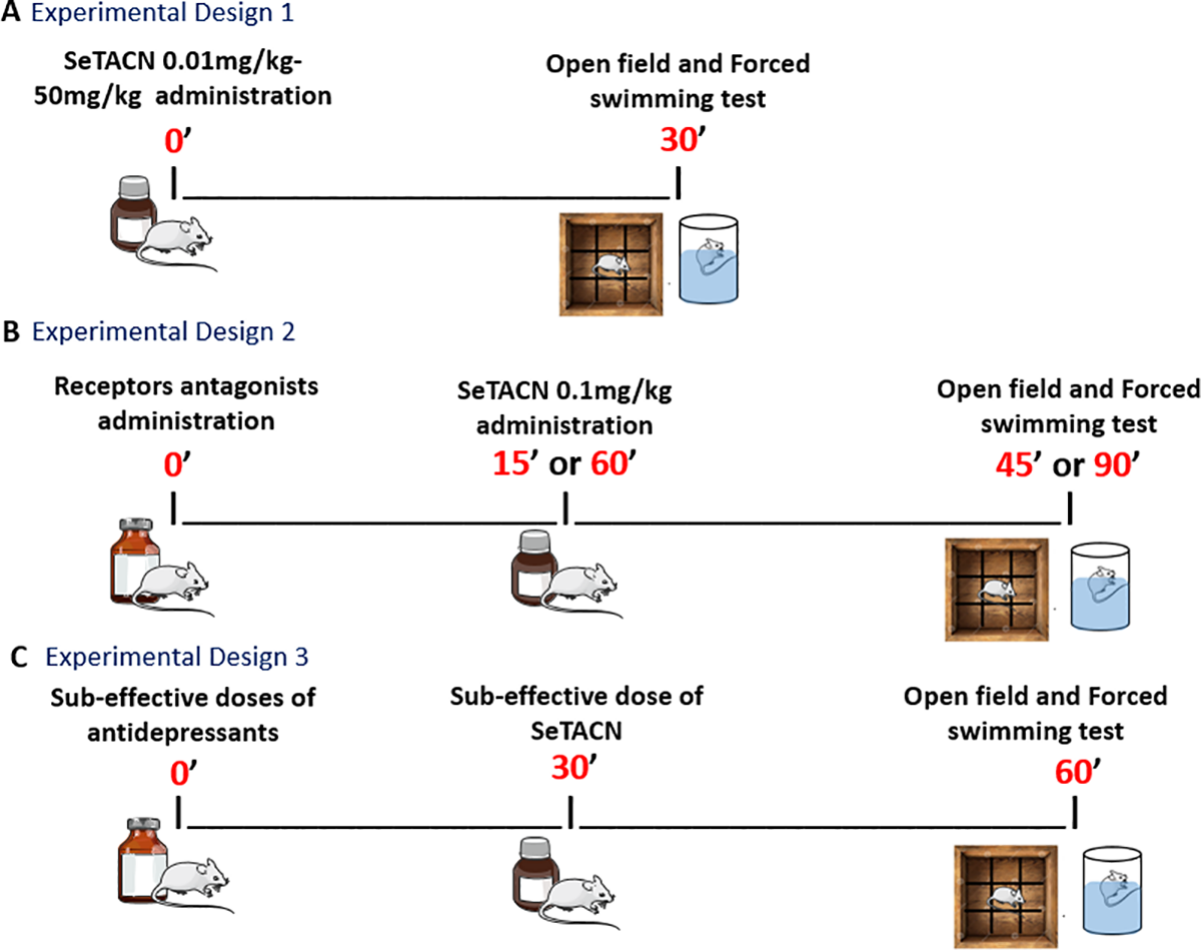
Experimental paradigms illustrating the drugs and compound administration followed by behavioral tests. (A) Antidepressant-like activity of 5-(4methoxyphenyl)-1-(2-(phenylselanyl)phenyl)-1H-1,2,3-triazole-4-carbonitrile (SeTACN). (B) Evaluation of mechanism of action involved in antidepressant-like effect of SeTACN. (C) Synergic effect of the combined treatment with sub-effective doses of clinical antidepressants and SeTACN.

We evaluated the antidepressant-like effect of resultant compound in mice submitted to forced swimming test (FST) too. For this purpose, the animals were treated with a dose range of SeTACN of 0.01mg-20mg/kg and 30 minutes later were submitted to open field test (OFT) and FST as can be seen in Fig 1A.

In view of investigate our *in silico* evidences about the compound mechanism of action, the animals were pretreated with different antagonists of monoaminergic receptors, in another set of experiments. After latency time for antagonist effect, the animals were treated with SeTACN (0.1mg/kg) and then submitted to OFT and FST (Fig 1B). The blockade of the SeTACN antidepressant-like effect by the administered antagonist is an indication of the involvement of this pathway.

We also evaluated the synergic effect of a sub-effective dose of clinical antidepressants with SeTACN, illustrated in Fig 1C. Combined effect of imipramine or fluoxetine and SeTACN in a synergistic antidepressant-like activity suggests that the antidepressant-like effect of SeTACN is attributed, at least in part, by a similar mechanism of action.

### 2.2 Homology modelling and molecular docking

The molecules analysed in this paper were drew using ChemDraw and their geometry optimized using the software Avogadro 0.9.4 following the MMFF94 method [31]. The molecular docking simulation was performed using software Autodock Vina [32], where all the rotatable bonds of ligands were allowed to rotate freely and the receptors were considered rigid.

Protein ligand interaction was observed by Autodock Tools [33]. Additionally, this software was used to minimize the structure of proteins, using the Gasteiger charges with 500 steps of minimization in all molecular targets.

We used crystallographic structures of molecular targets from Protein Data Bank (PDB) (http://www.pdb.org/). The CHIMERA 1.5.3 software was used to remove molecules, ions, and water [34].

**Fig 2:**
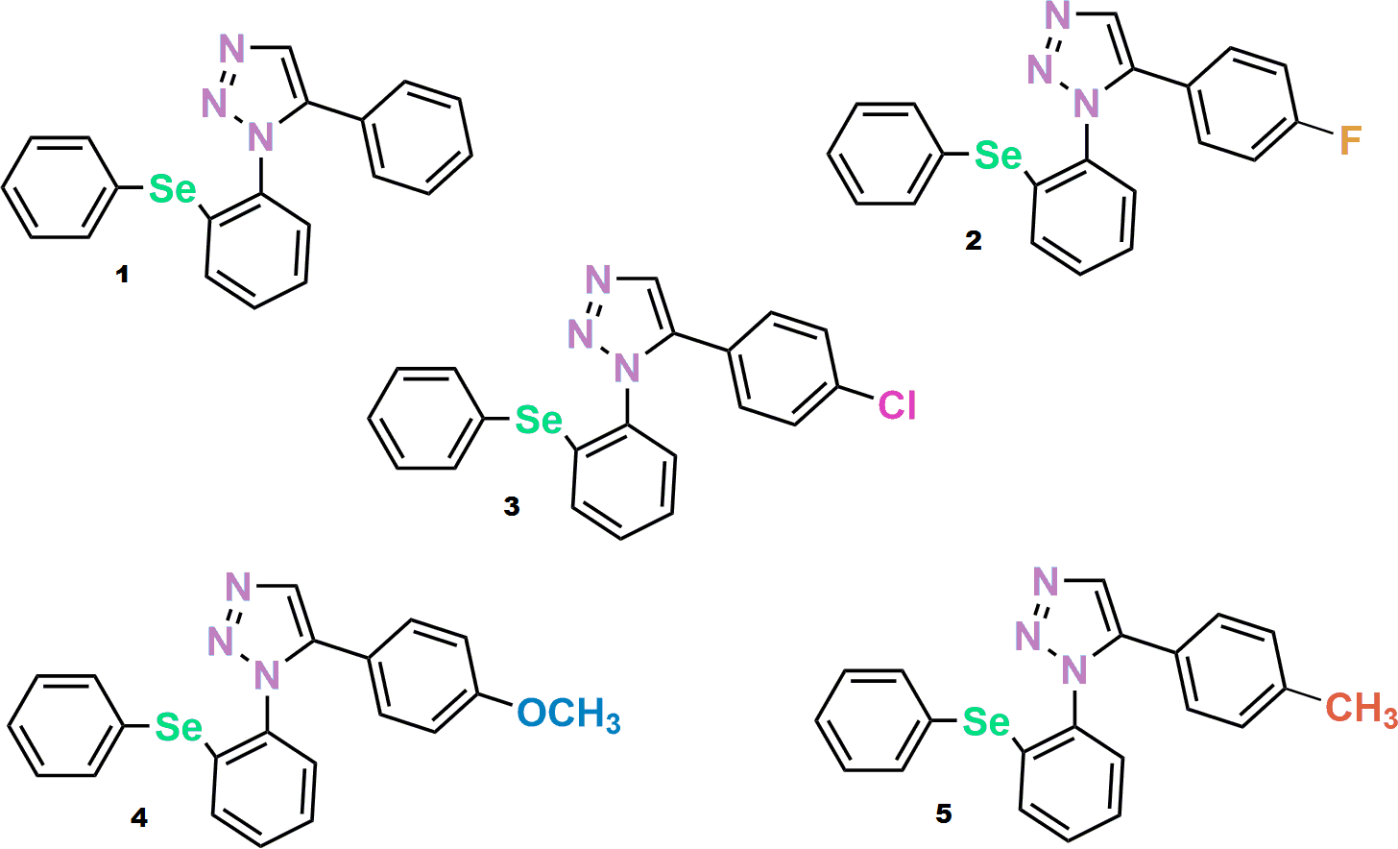
Chemical structure of class phenylselanyl-1*H*-1,2,3-triazole-4-carbonitriles compounds. Compound 1: 5-phenyl-1-(2-(phenylselanyl)phenyl)-1H-1,2,3-triazole-4-carbonitrile; Compound 2: 5-(4-fluorophenyl)-1-(2-(phenylselanyl)phenyl)-1H- 1,2,3-triazole-4-carbonitrile; Compound 3: 5-(4-chlorophenyl)-1-(2-(phenylselanyl)phenyl)-1H- 1,2,3-triazole-4-carbonitrile; Compound 4: 5-(4-methoxyphenyl)-1-(2-(phenylselanyl)phenyl)-1H- 1,2,3-triazole-4-carbonitrile and Compound 5: 1-(2-(phenylselanyl)phenyl)-5-(p-tolyl)-1H-1,2,3- triazole-4-carbonitrile.

Firstly, phenylselanyl-1H-1,2,3-triazole-4-carbonitriles (Fig 2) were docked in LeuBat (PDB:3GWV), protein LeuT with some mutations, being similar to SERT [35], a homology model. As positive controls, we used the molecules serotonin and fluoxetine.

Docking in dopamine transporter (DAT) (PDB:4M48) was performed using the same previously described methodology [36]. As serotonin is the major neurotransmitter involved in pathology of depression, the molecule with lowest docking score in DAT and highest docking score in SERT was selected for further investigation [37].

Additional studies were aimed to conduct docking in 5HT receptors 5HT_1a_, 5HT_2a_ e 5HT_3_. To reach this goal, the amino acid sequence of 5HT_1a_ was downloaded from UniProt database (accession code: P08908, 5HT1A_HUMAN) and the 3D structure of 5-HT1AR was constructed using the SWISS-MODEL server according to Zheng et al. (2015) [38]. 5HT_2a_ receptor was similarly built from 5HT_2b_ and the amino acid sequence of 5HT_2a_ Uniprot database (accession code: P28223) according to Gandhimathi and Sowdhamini, (2015) [39]. The structure utilized to perform the docking analyses was 5HT_3_ PDB: 4PIR requiring no homology studies.

### 2.3 Animals

The experiments were conducted using male *Swiss* mice (25–35 g, 60–75 days), housed in groups (3–5 animals per cage) under controlled conditions of light (7:00 to 19:00) and temperature (22–25°C). All tests were performed on separate groups of animals (n = 5–10) and each animal was used only once in each test. Before the start of the behavioral tests, the animals were allowed to acclimate in testing rooms for at least 1 hour. The behavioral analyses were performed by a blind measurer to the treatment conditions. Procedures of this study were conducted according to the guidelines of the Committee on the Care and Use of Experimental Animal Resources (NIH Publications No. 8023, revised 1978) and with the approval of the Ethical Comission for Animal Use of the Federal University of Pelotas, Brazil (7045–2015, process #23110.007045/2015-58). After treatment and behavioral analysis, mice were euthanized using a continue isoflurane flow. All efforts were made to minimize animals suffering and to reduce the number of animals used in tests.

### 2.4 Drugs

Ketanserin, ondansetron, sulpiride, SCH23390, p-chlorophenylalanine methyl ester (PCPA) and WAY100635 were purchased from Sigma Chemical Co, USA. Fluoxetine hydrochloride was purchased from Pfizer, Brazil and Imipramine hydrochloride was obtained from Novartis, Brazil. All these drugs were diluted in saline solution (0.9%) and injected via intraperitoneal (i.p) route, and WAY 100635 and SCH233390 administered via subcutaneous route (s.c). The commercial antidepressants were also diluted in saline solution (0.9%) but administered by intra gastric (i.g) route.

SeTACN was synthesized in our laboratory and characterized as previously described by Savegnago et al (2016) [23]. The compound was dissolved in canola oil and administered i.g. by gavage in mice. All the drugs listed were administered in a constant volume of 10 ml/kg body weight.

### 2.5 Behavioral tests

Based on the above mentioned *in silico* modelling, 5-(4methoxyphenyl)-1-(2-(phenylselanyl)phenyl)-1H-1,2,3-triazole-4-carbonitrile (SeTACN, Fig 1 –compound 4) was chosen for further analysis *in vivo*. This selection was based in SERT/DAT ratio best score, as determined by the logic created in this research.

In this way, in order to evaluate the antidepressant-like effect of SeTACN, the compound was administered once in mice (0.01-20mg/kg) and 30 minutes later, the animals were submitted to OFT followed by FST as experimental design 1 (Fig 1A).

#### 2.5.1 Open field test (OFT)

Locomotor activity was evaluated in the OFT, as previously described by Walsh and Cummings (1976) [40], to exclude a possible locomotor interference in FST. Briefly, animals were individually placed in a wooden square box (40 × 60 × 50 cm high) with 12 equal squares. The number of crossings were manually counted during a 5 minutes session. Crossing was considered only when animal crossed a line with four paws. After each session, the open field was cleaned with a solution of 70% ethanol to exclude any odor cues.

#### 2.5.2 Forced swimming test (FST)

FST was performed immediately after the OFT and was analyzed as previously described by Porsolt (1979) [41]. In summary, each mouse was individually placed in an open cylindrical container (diameter 10 cm, height 25 cm), with 19 cm of water at 25 ± 1°C, without the possibility of escaping, and was forced to swim. The total amount of time each animal remained immobile during 6 minutes session was recorded (in seconds) (only the last four minutes were analyzed). In this test, the immobile posture reflects a state of behavior despair and helplessness.

#### 2.5.3 Mechanisms involved in the antidepressant-like effect of SeTACN

The involvement of serotonergic system in the antidepressant-like effect of SeTACN (0.1mg/kg i.g.) was performed in another set of experiments included in experimental design 2 (Fig 1B). To reach this goal, mice were pre-treated with ketanserin (1mg/kg i.p.; a 5HT_2a_ receptor antagonist), ondansetron (1mg/kg i.p.; a 5HT_3_ receptor antagonist) or WAY100635 (0,1mg/kg s.c.; a 5HT_1a_ receptor antagonist) and 15 minutes later the animals were treated with a dose of SeTACN (0.1mg/Kg i.g.). After 30 minutes of compound administration, the animals were immediately exposed to OFT and FST.

With the purpose of verifying the influence of serotonin synthesis in antidepressant-like effect of SeTACN, animals were treated once a day with PCPA (100mg/kg, i.p., an inhibitor of serotonin synthesis) or vehicle (saline 0.9%) during 4 days. On the fifth day, animals received SeTACN (0.1mg/kg, i.g.) or just vehicle and 30 minutes later were submitted to OFT and FST.

The dopaminergic system involvement in antidepressant-like effect of SeTACN was verified according to experimental design 2 (Fig 1B). In this sense, animals were pre-treated with SCH23390 (0.05mg/kg, s.c., dopaminergic D_1_ antagonist receptor), sulpiride (50mg/kg, i.p., D_2_ receptor antagonist) or saline. After the 60 minutes, necessary for the antagonist effect, the animals were treated with SeTACN (0.1mg/kg) or vehicle. In the same manner as previously, FST and OFT were performed after 30 minutes of the compound administration. It is worth mentioning that, these methodologies were based in previous studies from Savegnago et al (2008) [42]; Martinez et al (2014) [43]; Pesarico et al (2014) [44] and Brod et al (2016) [24].

The effect of the co-administration of sub-effective doses of SeTACN (0.01mg/kg i.g.) and fluoxetine (5mg/kg, i.g., a selective serotonin reuptake inhibitor) was also investigated as predicted in experimental design 3 (Fig 1C) [45]. Thus, after 60 minutes of fluoxetine or vehicle administration the animals were treated with SeTACN or vehicle and after 90 minutes analyzed in the behavioral tests. The synergic effect of a sub-effective dose of SeTACN (0.01mg/kg) and imipramine (10mg/kg, i.g., a tricyclic antidepressant) was also evaluated (experimental design 3- Fig 1C) as mentioned above [46, 47].

### 2.6 Statistical analyses

The results were analyzed utilizing the software GraphPad Prism 5.0 and are given as the mean ± standard error of the mean (S.E.M.). Comparisons between experimental and control groups were performed by one-way or two-way analysis of variance (ANOVA) followed by Newman-Keuls test for post-hoc comparison when appropriate. Probability values less than 0.05 (P < 0.05) were considered as statistically significant.

## 3. Results and discussion

The molecular docking results in SERT and DAT are presented in Table 1. Based on this, the compound SeTACN (number 4) was chosen due to its higher score in SERT (-9,9kcal/mol) and lowest score in DAT (-9,0 kcal/mol). This rationale was developed based on studies which demonstrated that although SSRI have affinity for noradrenaline transporter (NET) and DAT, the SERT affinity is even higher [48, 49].

**Table 1:** Scores (kcal/mol) of docking results of phenylselanyl-1*H*-1,2,3-triazole-4-carbonitriles class of compounds in serotonin transporter (SERT) and dopamine transporter (DAT).

Compound	Docking in SERT (kcal/mol)	Docking in DAT (kcal/mol)
1	-8.3	-9.3
2	-9.8	-10.1
3	-10.0	-10.1
4	-9.9	-9.0
5	-10.1	-10.3

As positive controls in SERT, we utilized the molecules of 5-HT and fluoxetine, with a docking score of -7.1 and -8.7 respectively. In this way, the SeTACN affinity with SERT seems to be stronger, when its compared to 5-HT score, this data may indicate a preference in competitive binding to 5-HT transporter. This pattern is also observed when SeTACN score is compared to fluoxetine score, which might suggest a SeTACN stronger affinity to SERT, although more depth studies are required to affirm this hypothesis.

**Fig 3:**
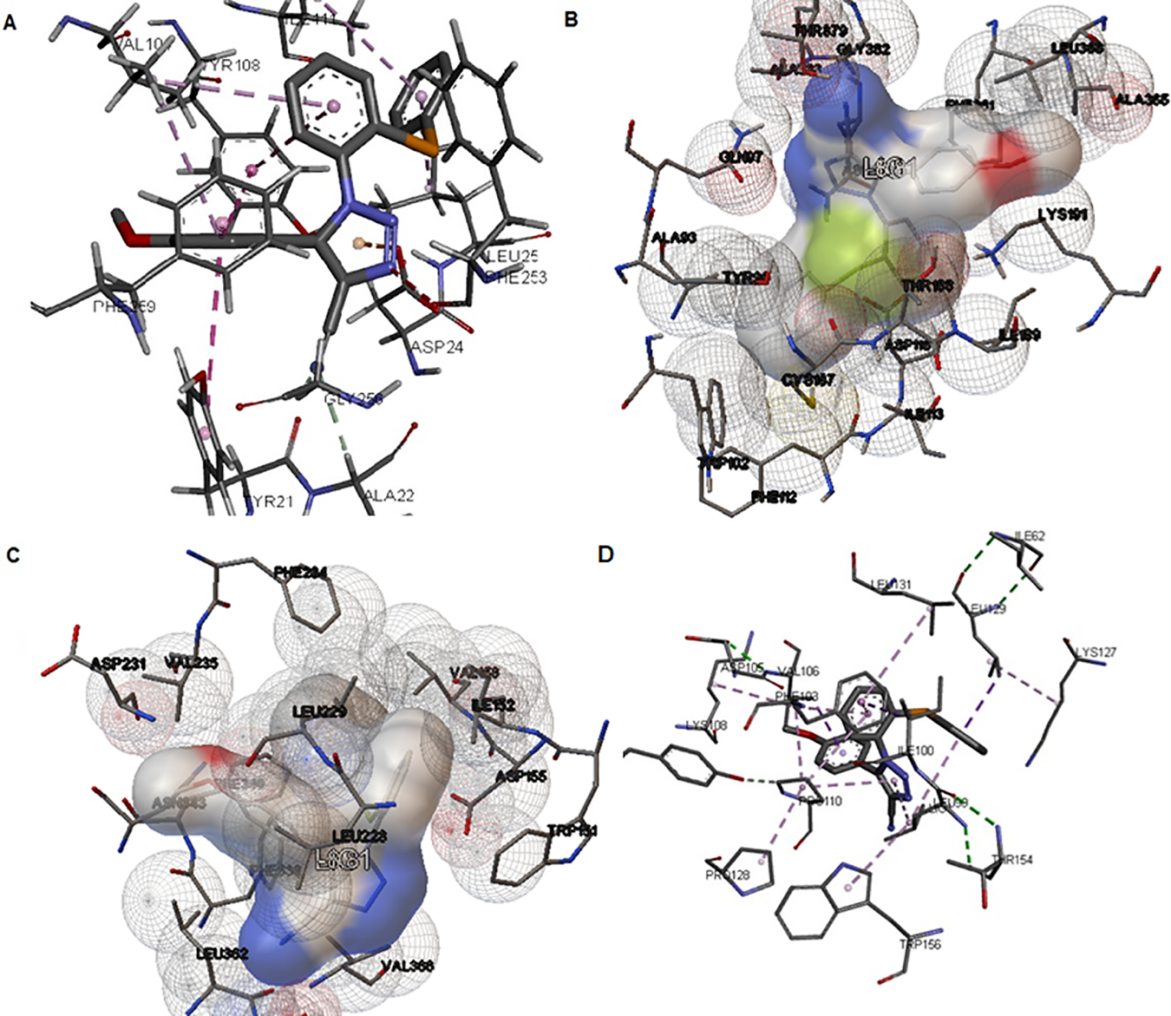
Docking results of compound 4 (5-(4methoxyphenyl)-1-(2- (phenylselanyl)phenyl)-1H-1,2,3-triazole-4-carbonitrile (SeTACN) in (A) serotonin transporter (SERT) with a score of -9.9kcal/mol (B) in 5HT_1a_ receptor with a score of -8.8kcal/mol (C) in 5HT_2a_ receptor with a score of -8.8kcal/mol (D) and in 5HT_3_ receptor with a score of -8.1kcal/mol.

SeTACN best score position is close to ASP 24 and TYR 21, which are target of paroxetine, sertraline and fluoxetine (Fig 3A). The residues PHE 259, VAL104, SER356 and TYR108 interaction of fluoxetine and sertraline is the same with SeTACN and leuBAT [50]. It is worth mentioning that the interaction of SeTACN with PHE253 an ASP404 might represent characteristic of specificity, similar to others SSRI [51].

The affinity of SeTACN with the serotonin receptor 5HT_1a_ is -8.8kcal/mol as shown in Fig 3B. The possible interaction with ILE113, PHE112, ASP116, ASN386, PHE361 and ALA365 are in agreement with some well-known 5HT1_a_ drugs as buspirone, 8-OH-DPAT and WAY100635 [52].

SeTACN docking score (-8.8 kcal/mol) in 5HT_2a_ and the nearest residues of the complex are illustrated in Fig 3C. The position of SeTACN in 5HT_2a_ receptors seems to be similar to antagonists of 5HT_2a_ such as espiperone, sharing the same residues interaction as TRP151, ILE152, LEU228, VAL156, ASP231 and PHE339 [37].

The result depicted in Fig 3D pointed out the docking scores of SeTACN in receptor 5HT_3_: -8.1kcal/mol. Although the score is lower when compared to other evaluated receptors, this interaction is considered significant. The best conformation of the compound is close to residues THR154 and TRP156, which inhibit this receptor by molecule VHH15 [53]. Moreover, the residue TRP156 is among those responsible for the opening and closing of 5HT_3_ ionic channels [53]. On the other hand, these residues interaction are not the same as antagonists like ondasetron and granisetron, which may suggest another way of 5HT_3_ inhibition [54].

**Fig 4:**
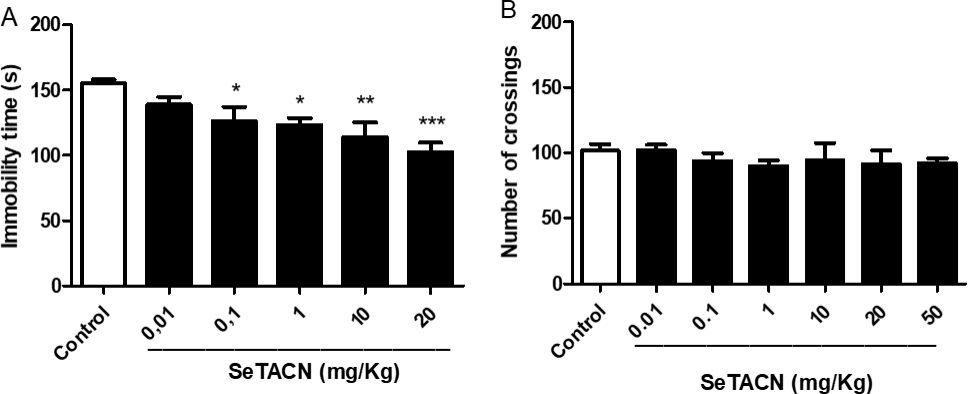
Effect of acute administration of SeTACN (0.01–20 mg/kg, i.g) in mice 30 min before (A) the forced swimming test (FST), and open field test (B). Values are expressed as mean S.E.M (one-way ANOVA followed by Newman Keuls) (*) P < 0.05, (**) P < 0.01, (***) P < 0.001 when compared to control group.

The SeTACN interaction with serotonergic system, explored by docking analyses suggests a possible antidepressant-like effect, which was explored under *in vivo* tests by FST. Results from Fig 4A indicate the effect of SeTACN on immobility time was statistically significant from 0.1–20mg/kg with respect to the control group (P< 0.05; P<0.01; P< 0.001). SeTACN given by i.g route and at all tested doses did not change the number of crossings in OFT when compared to the control group (Fig 4B). These findings pointed to a decrease in immobility time in FST not caused by any locomotor alteration.

**Fig 5:**
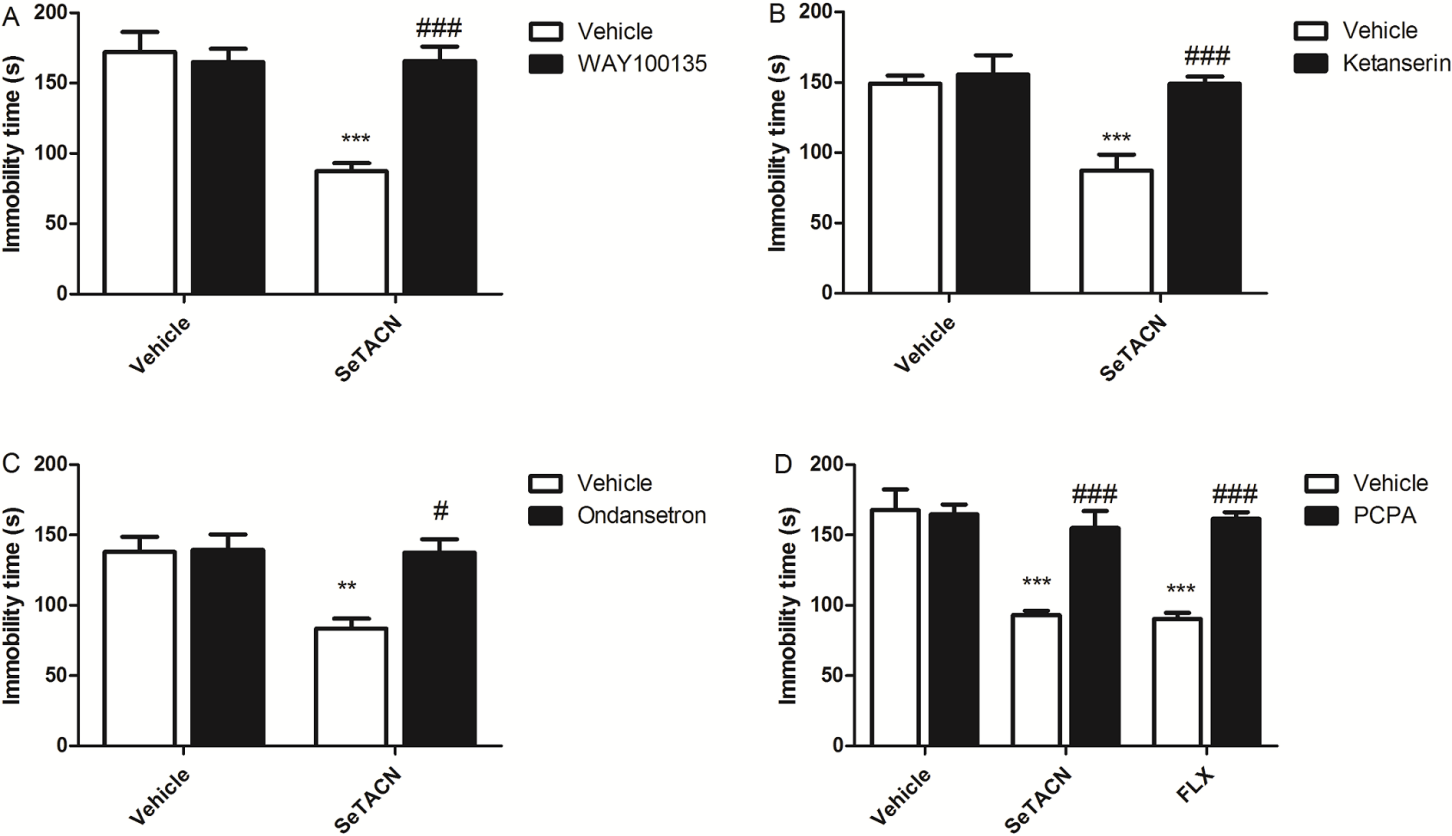
Effect of pretreatment of mice with (A) WAY100635 (0.1 mg/kg, s.c. a selective 5-HT1_A_ receptor antagonist); (B) ketanserin (1 mg/kg, i.p. a 5-HT_2A_ receptor antagonist); (C) ondansetron (1 mg/kg, i.p. a 5-HT_3_ receptor antagonist); and (D) PCPA (100 mg/kg, i.p., for 4 consecutive days, tryptophan hydroxylase inhibitor) on the anti-immobility effect of SeTACN (0.1mg/kg, i.g) in the FST. Data are presented as the mean ± S.E.M. (**) P < 0.01 and (***) P < 0.001 in comparison to the vehicle treated group (control); (#) P < 0.05 (###) P< 0.001 when compared to SeTACN pretreated with vehicle.

Fig 5A shows that pre-treatment with WAY100635 (a 5HT_1a_ receptor antagonist) was able to prevent the reduction of immobility time caused by SeTACN treatment (0.1mg/kg i.g). Two-way ANOVA analysis revealed a statistically significant effect of the treatment with SeTACN alone [F(1,23) = 16.64; P = 0.0005], WAY100635 alone [F(1,23) = 11.82; P = 0.0022], and treatment with WAY100635 x SeTACN [F(1,23) = 17.17; P = 0.0004]. No significant effect was observed for SeTACN treatment [F(1,23) = 0.04; P = 0.8523], WAY100635 treatment [F(1.23) = 0.05; P = 0.8284] or SeTACN × WAY100635 interaction [F(1,23) = 3.01; P = 0.961] on the number of crossings. These findings together with docking study 5HT_1a_ indicate the possible involvement of this receptor in the antidepressant-like effect of SeTACN.

The pre-treatment of mice with ketanserin (a 5HT_2a_ antagonist receptor) blocked the anti-immobility effect of SeTACN (0.1mg/kg) as demonstrated in Fig 5B, suggesting the involvement of 5HT_2a_. Two-way ANOVA tests revealed a statically significant effect of the treatment with SeTACN alone [F(1,27 = 13.95; P = 0.0009], ketanserin alone [F(1,27) = 13.84; P = 0.0009], and treatment with ketanserin x SeTACN [F(1,27) = 9.04; P = 0.0009]. No significant effect in OFT could be observed for SeTACN treatment [F(1,26) = 1.41; P = 0.2455], ketanserin treatment [F(1,26) = 0.85; P = 0.3639] or SeTACN × ketanserin interaction [F(1,26) = 0.01; P = 0.9321].

Results in Fig 5C demonstrate that pre-treatment with ondansetron (a 5HT_3_ receptor antagonist) could prevent the antidepressant-like effect of SeTACN (0.1mg/kg). Two-way ANOVA tests revealed significant differences in SeTACN treatment [F(1,16) = 8.41; P = 0.0105] and ondansetron × SeTACN treatment interaction [F(1,16) = 7.36; P = 0.0153] but not ondansetron treatment. No significant effect for SeTACN treatment [F(1,16) = 0.07; P = 0.8007], ondansetron treatment [F(1,16) = 0.01; P = 0.9329] or SeTACN × ondasentron interaction [F(1,16) = 0.47; P = 0.5935] was detected on the number of crossings.

This anti-immobility effect of SeTACN (0.1 mg/kg, p.o.) was blocked by the pre-treatment of mice with the inhibitor of serotonin synthesis, PCPA (Fig 5D). Two-way ANOVA showed main effect for SeTACN treatment [F(1,18) = 22.12, P = 0.0002] and PCPA × SeTACN treatment interaction [F(1,18) = 12.34, P = 0.0025] and also revealed significant differences for fluoxetine treatment [F(1,18) = 19.05; P = 0.0004], and PCPA × fluoxetine treatment interaction [F(1,18) = 16.15; P = 0.0008]. The two way ANOVA revealed no significant effect of SeTACN treatment [F(1,20) = 0.50; P = 0.4884], PCPA treatment [F(1.20) = 1.09; P = 0.3082] and SeTACN × PCPA treatment interaction [F(1,20) = 0.13; P = 0.7182] in number of crossings. No significant mobility effect for fluoxetine treatment [F(1,20) = 0.77; P = 0.3919] or fluoxetine × PCPA interaction either [F(1,20) = 0.73; P = 0.4041].

**Fig 6:**
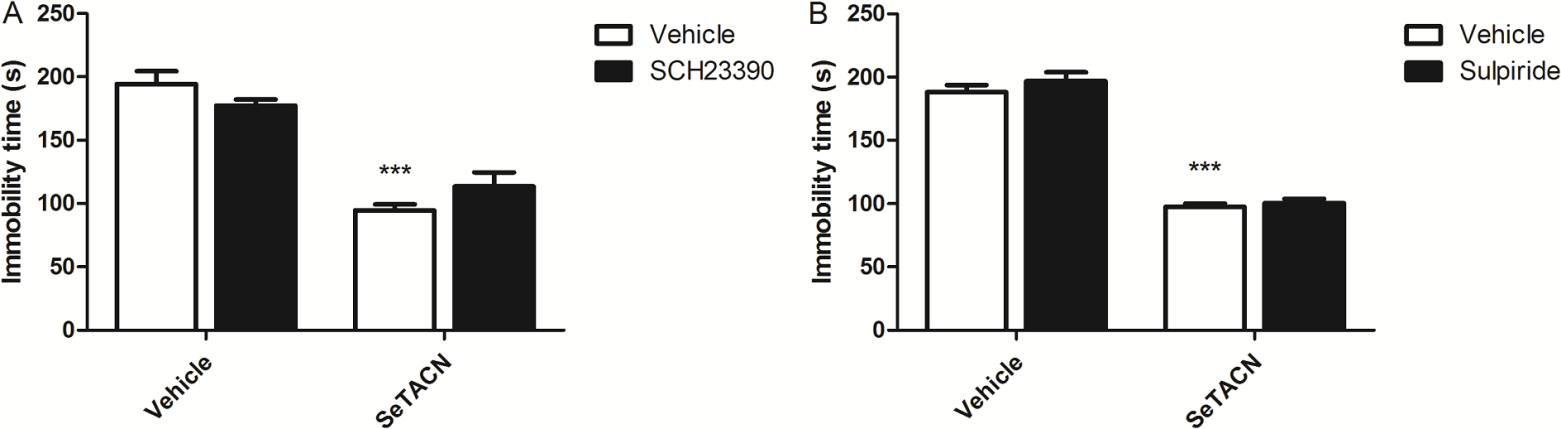
Effect of pretreatment of mice with (A) SCH233390 (0.05 mg/kg, s.c., a dopaminergic D_1_ receptor antagonist) and (B) sulpiride (50 mg/kg, i.p., a dopaminergic D_2_ receptor antagonist) on the anti-immobility effect of SeTACN (0.1mg/kg, i.g) in the FST. Data are presented as the mean ± S.E.M. (***) P < 0.001 in comparison to the vehicle treated group.

Interestingly, the pretreatment with SCH23390 (Fig 6A) or sulpiride (Fig 6B) did not block the antidepressant-like effect of SeTACN. Two-way ANOVA tests for immobility time revealed a main effect of SeTACN [F(1,29) = 85.32; P = 0.0001] and [F(1,18) = 395.10; P = 0.0001] respectively. The pretreatment of SCH23390 [F(1,29) = 4.06; P = 0.0533] and sulpiride [F(1,18) = 0.38; P = 0.5449] did not eliminate the antidepressant-like effect elicited by SeTACN. Two-way ANOVA of OFT showed that SeTACN treatment did not produce any significant effect in mice locomotor activity [F(1,29) = 1.65; P = 0.2092], SCH23390 treatment [F(1,29) = 2.08; P = 0.1595] and SeTACN × SCH23390 treatment interaction [F(1,29 = 1.33; P = 0.2583] with respect to number of crossings. In the same way, no significant effect was observed for SeTACN treatment [F(1,16) = 0.13; P = 0.7258], sulpiride treatment [F(1,16) = 0.08; P = 0.7755] or SeTACN × sulpiride interaction [F(1,16) = 0.51; P = 0,4855]. These results suggest that the antidepressant-like effect of SeTACN may not be influenced by the D1 or D2 receptors, but more studies in relation to dopaminergic system and SeTACN are necessary.

**Fig 7:**
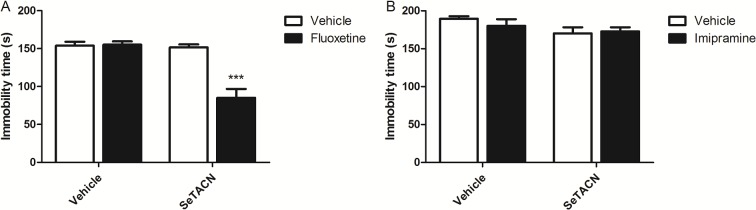
Co-administration of sub-effective doses of (A) fluoxetine (5 mg/kg, i.g) or (B) imipramine (10 mg/kg, i.g) and (SeTACN 0.01 mg/kg, i.g) in the immobility time FST. Values are expressed as mean ± S.E.M. (***) P < 0.001 in comparison to the vehicle treated group.

Fig 7A summarizes the synergetic effect between immobility time of animals treated with a sub-effective dose of fluoxetine (5mg/kg; selective serotonin reuptake inhibitor) in combination with a sub-effective dose of SeTACN (0.01mg/kg). Two-way ANOVA tests revealed no effect of the treatment with SeTACN alone [F(1,12) = 26.30; P = 0.0002], fluoxetine alone [F(1,12) = 21.45; P = 0.0006], and treatment with fluoxetine x SeTACN [F(1,12) = 23.13 P = 0.0004]. No significant effect for SeTACN treatment [F(1,12) = 0.12; P = 0.7398], fluoxetine treatment [F(1,12) = 0.68, P = 0.4252] or SeTACN × fluoxetine interaction [F(1,12) = 0.01, P = 0.9117] was observed with respect to the number of crossings. These findings imply that fluoxetine and SeTACN may have a similar mechanism of action.

However, the effect between a sub-effective dose of imipramine (10mg/kg; a tricyclic antidepressant) and SeTACN (0.01mg/Kg) was not significant in immobility time (Fig 7B). Two-way ANOVA tests revealed the SeTACN effect alone [F(1,19) = 3.80; P = 0.0660], imipramine effect alone [F(1,19) = 0.28; P = 0.6039] and the combination of SeTACN × imipramine treatment interaction [F(1,19) = 0.78; P = 0.3878]. Either, in open field test of SeTACN treatment [F(1,19) = 0.03; P = 0.8718], imipramine treatment [F(1,19) = 0.01; P = 0.9441] or SeTACN × imipramine interaction [F(1,19) = 0.04; P = 0.8479] did not change the mice locomotor activity.

Taken together, the results in the present study, both computational and behavioral, suggest that the antidepressant-like effect of SeTACN in FST depends on the interaction of serotonergic neurotransmission. Probably, firstly due the inhibition of SERT and as a complementary action, the modulation of 5HT-receptors as 5HT_1a,_ 5HT_2a_ and 5HT_3._ Besides, SeTACN is capable of restoring the despair behavior induced by PCPA which lead to a serotonin depletion, this data may infer the modulation of 5HT synthesis. Furthermore, based in this preliminary evaluation, is important to highlight the hyphothetical feature of this mechanism of action exerted by SeTACN and more studies are required to support these evidences.

The suggested complementary SERT mechanism of action, trough the modulation of 5HT receptors could be benefic in depression pathogenesis. Since, 5HT_1a_ autoreceptors are responsible in the self-inhibition control of 5HT neurons [55]. Most antidepressant drugs increase the concentration of 5HT in the extracellular brain space only by preventing its reuptake trough the blockade of SERT [56]. Indeed, this increase is offset by a negative feedback operating at the 5HT_1a_ autoreceptors. This mechanism is thought to be responsible for the delay in onset of the therapeutic action, often by several weeks, of antidepressants [6]. In this sense, compounds which interact in 5HT_1a_ receptors can accelerate the antidepressant response to SSRIs, acting by potentiating 5HT neurotransmission [57–59].

In addition, preclinical studies indicate that 5HT_2a_ receptor subtype represent a promising target in SSRIs-resistant depressive patients, potentiating the behavioral effects of SSRIs [60]. Besides, the stimulation of 5HT_2a_ receptors is related direct and indirectly to the modulation of adult neurogenesis in the hippocampus and antidepressants exert their therapeutic activity, at least in part, by stimulating this pathway [61].

5HT_3_ receptors also have a critical influence on behavioral and neurocircuitry processes in brain that control mood and emotional behavior [9]. It is well known that the mechanism of action of fluoxetine and other antidepressants, are related to the non-competitive antagonism of the 5HT_3_ receptor [62]. Moreover, another interesting characteristic of 5HT_3_ receptors is the presence of chemoreceptor trigger zone in brainstem and in the gastrointestinal tract, which mediate nausea/vomiting motility, which may protect against the gastrointestinal side effects that often accompany SSRIs antidepressants [12].

Interesting, triazole is the core structural motif exhibits a broad range of biological properties, including antidepressant-like activity as previously reported [63–65]. This nucleus is also present in antidepressant drug Nefazodone, which generates its therapeutics effects primarily as potent 5HT_2a_ inhibitor. Besides, has moderate effects as 5HT_1a_ inhibitor and serotonin-norepinephrine-dopamine reuptake inhibitor (SNDRI) through the interaction with monoaminergic transporters [66; 67].

The computational tools, such as molecular docking has contributing to drug design, in the discovery of new molecules with therapeutic effects and contributing to suggest its mechanism of action as well [68]. In this way, this study shows for the first time a selenium compound binding affinity with serotonin transporters and the serotonin receptors: 5HT_1a,_ 5HT_2a_ and 5HT_3_ which might be useful to unravel the mechanism of action antidepressant-like effect exerted by several selenium compounds, as cited previously_._ Moreover, similar studies already demonstrated the antidepressant-like effect using this docking methodology in mice submitted to FST [69–71].

FST is one of the most used tools for antidepressants screening, in this sense, a reduction in immobility time is considered indicative of an antidepressant-like effect [72; 73]. Although, we can just suggest a possible antidepressant-like activity of SeTACN, because a current limitation of this study is the absence of an induced depressive-like behavior in mice. Considering, future studies are needed to conclude the mechanism of action and determine the antidepressant clinical efficacy of SeTACN.

Another interesting characteristic of SeTACN is the antioxidant effect in mice cerebral cortex and hippocampus, already demonstrated by our research group [23]. Studies demonstrated that depressed patients present a reduction in volume and function of these areas [74, 75]. These structural changes happen due the atrophy of several dysregulated signaling pathways, including oxidative stress [76, 77]. In this sense, the antioxidant effect of SeTACN could at least in part diminishes the negative impact of the redox dysregulation in neuronal homeostasis.

In this context, some antidepressants already demonstrated antioxidant effects and other antioxidants have been reported to exert antidepressant-like effect [78; 79]. So, the antioxidant potential of SeTACN could contribute to its antidepressant-like effect. Despite, it is just a hypothesis and to more concrete conclusions further studies are needed regarding the antioxidant role in antidepressant-like effect of SeTACN.

Behavioral findings and molecular studies have shown that different subtypes of 5HT receptors might relate to the effectiveness of the antidepressant compounds [80]. Taking all these data, we can suggest that SeTACN might be a antidepressant-like compound with an interest hypothetical mechanism of action, blocking the SERT and with affinity to 5HT_1a_, 5HT_2a_ and 5HT_3_. This mechanism could accelerate the onset of action and diminishes others side effects of the current prescribed antidepressants. Although more studies are needed to affirm SeTACN pharmacological antidepressant efficacy.

## 4. Conclusion

In conclusion, based on computational and behavioral evidence, SeTACN exerted antidepressant-like activity in mice, through the possible modulation of serotonergic pathway. Nevertheless, further studies are needed to elucidate the mechanism of action and the contribution of other neurotransmission systems, signaling pathways using others depressive models and experimental techniques.

## Supporting information

S1 Graphical abstract Scheme illustrating the methodology performed in view of explore the antidepressant-like activity of SeTACN. (TIF)
