# Correction: Computational and biological evidences on the serotonergic involvement of SeTACN antidepressant-like effect in mice

**DOI:** 10.1371/journal.pone.0189975

**Published:** 2017-12-14

**Authors:** Mariana G. Fronza, Lucimar M. Pinto Brod, Angela Maria Casaril, Manoela Sacramento, Diego Alves, Lucielli Savegnago

Fig 2 is incorrect. The authors have provided a corrected version here.

**Fig 2:**
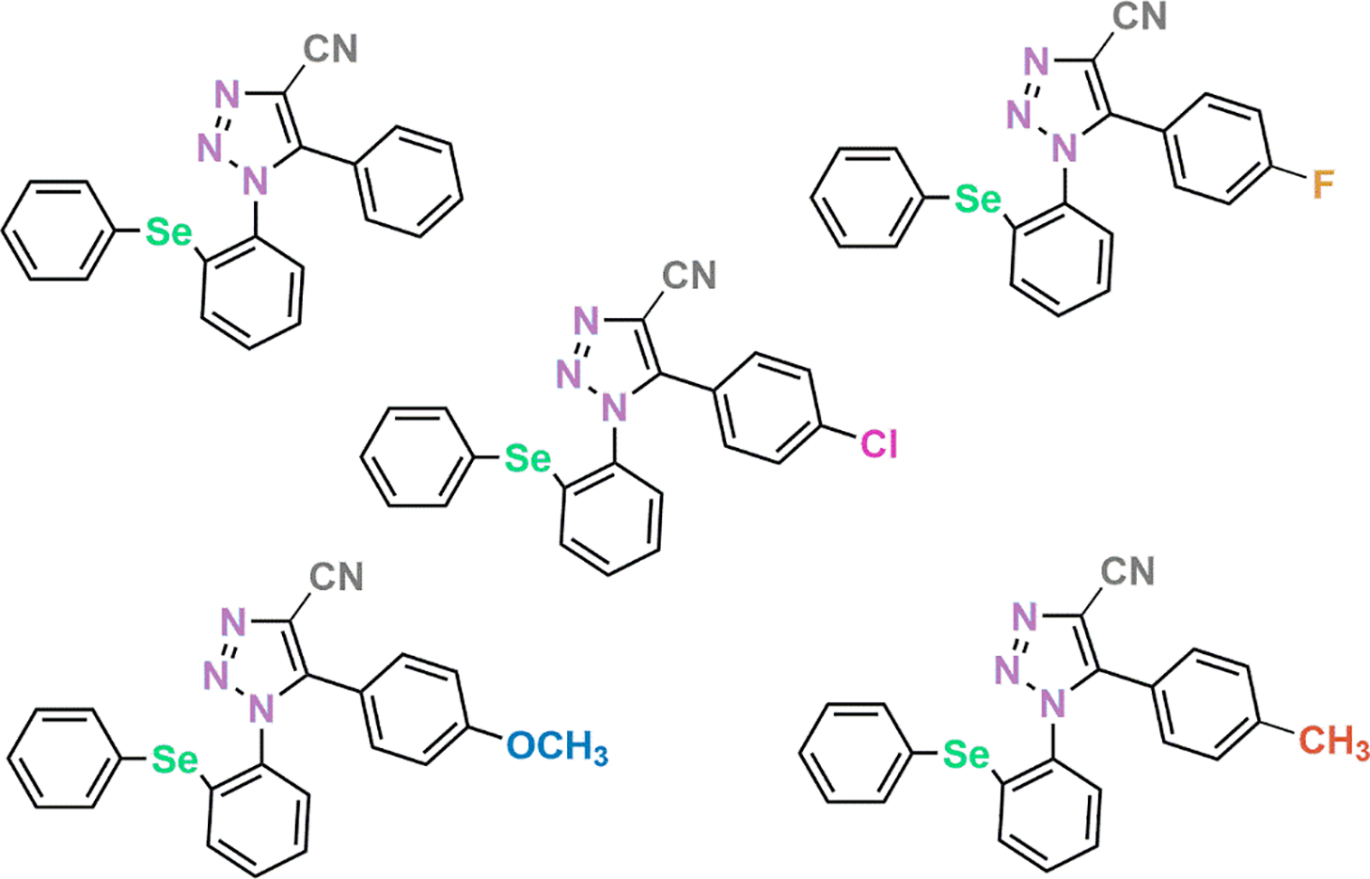
Chemical structure of class phenylselanyl-1H-1,2,3-triazole-4-carbonitriles compounds. Compound 1: 5-phenyl-1-(2-(phenylselanyl)phenyl)-1H-1,2,3-triazole-4-carbonitrile; Compound 2: 5-(4-fluorophenyl)-1-(2-(phenylselanyl)phenyl)-1H- 1,2,3-triazole-4-carbonitrile; Compound 3: 5-(4-chlorophenyl)-1-(2-(phenylselanyl)phenyl)-1H- 1,2,3-triazole-4-carbonitrile; Compound 4: 5-(4-methoxyphenyl)-1-(2-(phenylselanyl)phenyl)-1H- 1,2,3-triazole-4-carbonitrile and Compound 5: 1-(2-(phenylselanyl)phenyl)-5-(p-tolyl)-1H-1,2,3- triazole-4-carbonitrile.
