# Supplementary figures and images for: Computational and biological evidences on the serotonergic involvement of SeTACN antidepressant-like effect in mice
Source: PLoS One. 2017 Nov 1;12(11):e0187445. doi: 10.1371/journal.pone.0187445 (PMC5665604; doi:10.1371/journal.pone.0187445)

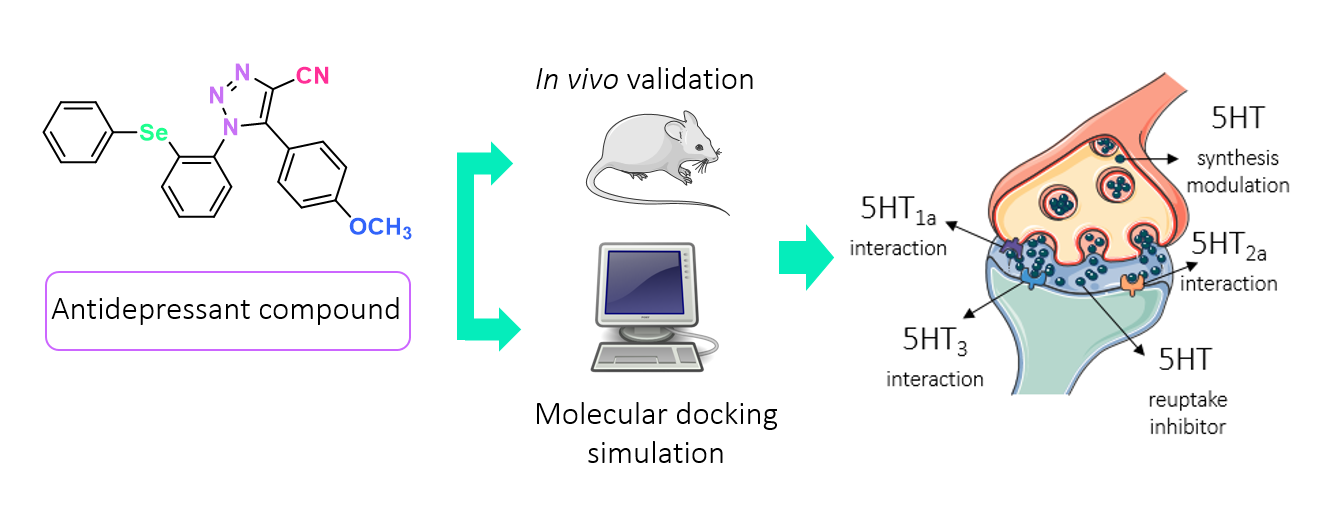

Supplement: S1 Graphical abstract — (TIF) [file pone.0187445.s001.tif]
